# Use of Femoral and Sciatic Nerve Block Combination in Severe Emphysematous Lung Disease for Femoral Popliteal Arterial Bypass Surgery

**DOI:** 10.7759/cureus.2140

**Published:** 2018-02-02

**Authors:** Onur Selvi, Olgar Bayserke, Serkan Tulgar

**Affiliations:** 1 Aneasthesiology and Reanimation, Maltepe University Faculty of Medicine; 2 Cardiac and Vascular Surgery, Maltepe University Facullty of Medicine; 3 Anaesthesiology and Reanimation, Maltepe University Faculty of Medicine

**Keywords:** vascular surgery, nerve block

## Abstract

Regional anesthesia is a widely used anesthesia technique for high-risk patients with severe vascular or pulmonary diseases in which general anesthesia is considered harmful and should be avoided. In this case, we present the use of femoral-sciatic nerve block combination for a 65-year-old, ASA IV, male patient who had severe emphysematous lung disease and was planned for a right femoral-popliteal arterial bypass surgery. He had severe pulmonary disease, hypertension, peripheral vascular disease, and was on clopidogrel treatment. Due to his existing comorbidities, regional anesthesia was considered the right method. The combination of femoral and sciatic nerve block was successfully used for the operation, which lasted for one hour and fifty minutes under sedation, and was continuously supplied with a propofol infusion. The patient was safely discharged to the surgical ward with no pain. The femoral block and sciatic block combination is described as one of the most useful, and at the same time, the most ignored anesthetic method. This technique is considered a standard technique and is often taught early in training; however, its use seems to be underestimated as there are only a few cases documented in Turkey. The aim of this case is to serve as a reminder of its significant value in patients who are not appropriate for general anesthesia and neuraxial blocks.

## Introduction

Regional anesthesia techniques are used for varying reasons including postoperative analgesia, surgical procedures, and chronic pain treatment with a single injection or continuous methods. They can be a convenient choice for surgeries of complicated high-risk patients with vascular and cardiopulmonary diseases in which general anesthesia may deteriorate their current delicate status [1]. Regional anesthesia, preferably in a combined form, could be a secure choice for this group of patients. We present the case of a high-risk patient with coronary problems, in addition to severe pulmonary disease and history of acute respiratory distress syndrome (ARDS), who was planned for femoro-popliteal bypass under the combined femoral-sciatic block.

## Case presentation

A 65-year-old, ASA IV, 40-kg (body mass index (BMI) 22.5) male patient with severe emphysematous lung disease presented for a right femoral-popliteal arterial bypass surgery after informed consent was obtained from the patient for inclusion into this report. His medical history was significant for a 90 pack-year of cigarette smoking, restrictive pulmonary disease, hypertension, and peripheral vascular disease. The patient had undergone coronary artery bypass grafting (CABG)x6 surgery seven months ago. In the postoperative period, he was accepted to the intensive care unit (ICU) with the diagnosis of ARDS. He had mechanical ventilation support with tracheostomy for a month. Unfortunately, on the 28th day of his stay in the ward, he had a sudden cardiac arrest and was successfully resuscitated. After a month-long treatment in the ICU, he was discharged to the general ward with no neurological deficits. His tracheostomy was surgically closed three months ago. Two weeks before the femoral-popliteal arterial bypass surgery, he had interventional bilateral iliac stents placement and was discharged home on clopidogrel therapy.

He was on low molecular weight heparin, metoprolol, lansoprazole, alprazolam medications, and had a normal electrocardiogram (ECG). He had grade I left ventricle diastolic dysfunction with 60% ejection fraction (EF). His laboratory data were within normal limits except for albumin (2.7 g/dL) and albumin and hemoglobin (9.3 mg/dL). ECG, pulse oximetry (SaO2), and invasive blood pressure measurement were monitored. The patient was sedated with 50 ugr fentanyl, 2 mg midazolam, and 25 mg ketamine. The SpO2 level was 97%-99% under 5 ml/min oxygen therapy. The nerve block solution was prepared with 20 ml 0.5% bupivacaine, 10 ml 2% lidocaine, 10 ml NaCl 0.9% for the femoral and the sciatic blocks; the adequate anesthesia was achieved after 30 minutes of the required level for the operation. The operation lasted one hour and fifty minutes under sedation with propofol infusion and the patient was safely discharged to the surgical ward with no pain.

## Discussion

General anesthesia may cause shunts or atelectasis in the lungs, which may deteriorate existing pulmonary pathologies [2]. These changes may not be tolerated in patients with severe comorbidities. Regional anesthesia may let high-risk patients have safer anesthesia devoid of any pulmonary and cardiovascular adverse changes. Chronic arterial occlusion is a pathology which may need revascularization therapies [1]. These operations can be performed under general, spinal, or epidural anesthesia. However, these patients show hypercoagulopathy with high prevalence rate [3].

Regional blockade techniques are found superior to general anesthesia regarding postoperative analgesia and suppression of the response to surgical stress leading to hypercoagulability. However, in most cases, patients receive anticoagulant or thrombolytic medications which question the safety of spinal and epidural anesthesia [1,4]. The European Society of Anaesthesiology recommends using superficial blocks such as axillary femoral or distal sciatic nerve blocks which may be applied to anticoagulant therapies. Meanwhile, the time intervals for low-molecular-weight heparin (LMWH) administration and catheter manipulations are recommended to be at least 12 hours, as followed in our case [5].

The analgesic effects of combined femoral and sciatic blocks have been well documented in the literature and can be still a good choice for anaesthesiologists who work in a difficult environment such as poor postoperative care service or limited numbers of staff members. This combination provides 12-13 hours postoperative analgesia which can be extremely beneficial without facing the risks of general anesthesia-related complications in such conditions [6-7]. Although continuous spinal and epidural anesthesia has superior analgesia and better hemodynamic stability, patients who are not eligible for neuraxial blocks due to their anticoagulant medication can safely undergo lower extremity and inguinal surgeries with combined sciatic and femoral block [7].

We were able to supply rapid onset of anesthesia by using lidocaine which is a less toxic substance than bupivacaine. We used lidocaine and bupivacaine combination in lower doses; however, it was applied in an increased volume. The lower concentration of anesthetic agents can be helpful in preventing side effects of these drugs while enlarged volume expands the analgesic cover [8].

## Conclusions

The femoral block and sciatic block combination is described as one of the most useful, at the same time, the most ignored regional technique. This case report is one of the first published reports of combined femoral and sciatic nerve block for femoral-popliteal bypass surgery in Turkey, and this fact reveals the underestimated value of this block. Although this technique is considered a standard technique and is often taught early in the training of anaesthesiologists, it might have been ignored in daily practice. The purpose of this case presentation may remind us to value it properly.
